# Disruption of Neural Activity and Functional Connectivity in Adolescents With Major Depressive Disorder Who Engage in Non-suicidal Self-Injury: A Resting-State fMRI Study

**DOI:** 10.3389/fpsyt.2021.571532

**Published:** 2021-06-01

**Authors:** Qian Huang, Muni Xiao, Ming Ai, Jianmei Chen, Wo Wang, Lan Hu, Jun Cao, Mengyao Wang, Li Kuang

**Affiliations:** ^1^Department of Psychiatry, The First Affiliated Hospital of Chongqing Medical University, Chongqing, China; ^2^Mental Health Center, University-Town Hospital of Chongqing Medical University, Chongqing, China

**Keywords:** major depressive disorder, non-suicidal self-injury, amplitude of low-frequency fluctuation, resting-state functional connectivity, visual regions, default mode network

## Abstract

**Background:** Non-suicidal self-injury (NSSI), which commonly occurs during adolescence, often co-occurs with major depressive disorder (MDD). However, the underlying neurobiological mechanisms in adolescents with MDD who engage in NSSI remain unclear. The current study examined the aberrant local neural activity in certain areas of the visual regions and the default mode network (DMN) and the resting-state functional connectivity (rs-FC) in changed brain regions in adolescents with MDD who engage in NSSI and adolescents with MDD only.

**Methods:** A total of 67 adolescents with MDD were divided into two groups based on their NSSI behavior: the NSSI group (*n* = 31) and an age-, gender-, and education-matched MDD group (*n* = 36). The Hamilton Depression Rating Scale (HAMD) was used to assess the severity of MDD. Amplitude of low-frequency fluctuation (ALFF) analysis was used to detect alterations in local neural activity. Brain regions with aberrant neural activity were considered regions of interest (ROI). ALFF-based rs-FC analysis was used to further explore the underlying changes in connectivity between ROI and other areas in the NSSI group. Correlation analyses were performed to examine the relationship between neural changes and clinical characteristics.

**Results:** There was no significant difference in HAMD scores between the two groups. ALFF analysis revealed that, compared to adolescents with MDD only, adolescents with MDD who engaged in NSSI displayed significantly enhanced neural activity in the right fusiform gyrus (FFG. R) and the right median cingulate and paracingulate gyri (DCG. R). Significantly reduced rs-FC of the FFG. R-bilateral medial orbital of the superior frontal gyrus (ORBsupmed. L/R)/bilateral medial superior frontal gyrus (SFGmed. L/R), FFG. R-bilateral posterior cingulate gyrus (PCG. L/R), DCG. R-left pallidum (PAL. L), DCG. R-right superior temporal gyrus (STG. R), and DCG. R-right postcentral gyrus (PoCG. R)/right inferior parietal lobule (IPL. R) was found in adolescents with MDD who were engaged in NSSI. Additionally, no significant correlations were observed between ALFF or rs-FC values and the HAMD scores between the two groups.

**Limitations:** Owing to the cross-sectional design, the alterations in ALFF and rs-FC values in the FFG. R and DCG. R could not demonstrate that it was a state or feature in adolescents with MDD who engaged in NSSI. Additionally, the sample size was relatively small.

**Conclusions:** This study highlights changes in regional brain activity and remote connectivity in the FFG. R and DCG. R in adolescents with MDD who engage in NSSI. This could provide a new perspective for further studies on the neurobiological mechanism of NSSI behavior in adolescents with MDD.

## Introduction

Major depressive disorder (MDD) is the most prevalent mental disorder and is mainly characterized by aberrant emotion (lack of pleasure) and cognitive function (dysfunctional attention and memory) (1). MDD is a widely accepted risk factor for suicide attempts. Up to 50–70% of suicide victims may be affected by MDD (2). Adolescence is a pivotal period during which considerable changes occur in physiological, psychological, and social relationships (3). Adolescent individuals are highly vulnerable to mental disorders and maladaptive behaviors, including MDD and non-suicidal self-injury (NSSI). NSSI, which refers to directly and deliberately damaging one's own body tissue without intending to end one's life (4), often occurs in the context of a diagnosis such as borderline personality disorder [BPD; (5)], MDD (6) or other psychiatric disorders. In addition, it can also occur independently of a psychiatric diagnosis. NSSI, as a common method of regulating stress, is generally used by adolescents to improve interpersonal difficulties and regulate negative emotions (7). The prevalence of adolescents who engage in NSSI has been estimated to be 17% in community samples (8) and more than 40% in clinical samples (9). Prior research has demonstrated that NSSI is a crucial precursor for future suicidal behavior (10, 11). Therefore, adolescents with MDD who engage in NSSI may have a higher risk of suicidal behavior. Although NSSI has gradually attracted attention from scholars owing to its high incidence rate and high risk of suicide in adolescence, its neurobiological mechanism remains unclear.

Facial expressions are one of the best methods for mapping an individual's emotional status. Visual regions play an important role in facial expression recognition and visual information processing when processing emotional facial stimuli (12). Functional magnetic resonance imaging (fMRI) studies have shown that functional changes in the visual regions are significantly associated with MDD (13–15). Alterations in the visual pathway involving the middle occipital gyrus and downstream regions (e.g., the fusiform gyrus [FFG]) have been reported in MDD during visual categorization (16, 17), attention (14), and working memory tasks (13). Significantly enhanced activity in the occipital gyrus has been reported in patients with MDD in a task-based fMRI study (18). In addition, neural pattern aberrations in the occipital gyrus were also found in individuals with NSSI while watching emotional and NSSI images (19). The FFG, a vital region of the high-level visual cortex, has been thoroughly researched in recent years because of its abnormal structure and function in patients with mental disorders. A previous study showed that the FFG may be associated with cognitive processing in patients with MDD (20). Increased amplitude of low-frequency fluctuation (ALFF) in the FFG and decreased resting-state functional connectivity (rs-FC) and resting-state global functional connectivity density between the FFG and other regions were observed in patients with MDD (21, 22). Moreover, research also reported that, compared to patients with MDD without NSSI, MDD youth with a history of NSSI had an enhanced response in the FFG after completing an interpersonal self-processing task from their mother's perspective using fMRI, which indicated that FFG activation may be associated with the intense socioemotional processing of NSSI youth (23). Although this evidence has provided crucial clues to the potential neurobiological underpinning of MDD or NSSI, to the best of our knowledge, there are few neuroimaging studies on patients with MDD with NSSI, despite the high prevalence rate and high risk of suicide, let alone in adolescents. Furthermore, there are no rs-fMRI studies on the neural function of visual regions in adolescents with MDD who engage in NSSI. Hence, the neural function of the visual regions is one of the focuses of the current study.

Brain networks that are effectively used for the segregation and integration of information processing have been extensively explored in the past decade. Many disrupted neural networks in MDD have been reported; however, the pervasively concerned networks are the default mode network (DMN), salience network, and central executive network (24–26). These networks are obviously connected with emotional regulation and cognitive processing in patients with MDD; therefore, changes in neural networks that contribute to emotional disorders in patients with MDD might also negatively influence cognitive function. The DMN, which is linked to rumination, has received the most attention (27). The DMN is divided into two components. The key regions of the anterior DMN are the medial prefrontal cortex (MPFC) and ventral anterior cingulate cortex, and the key regions of the posterior DMN are the precuneus/posterior cingulate cortex (PCC) and bilateral angular gyrus (28, 29). Berman et al. demonstrated that rumination in patients with MDD correlates with increased rs-FC between the PCC and the subgenual cingulate cortex (30). A meta-analysis of rumination in patients with MDD demonstrated that rumination has a positive relationship with the rs-FC between the DMN (especially the PCC) and the subgenual prefrontal cortex (27). Rumination, as one of the core symptoms of MDD, not only is related to vulnerability to the onset and relapse of MDD, but also facilitates the maintenance of negative mood (31). It is also an effective predictor of NSSI because it regulates emotions in NSSI (32). Prior evidence suggests that it may be an effective emotion regulation method to mediate the relationship between NSSI behavior and depressive symptoms (33, 34). Several neuroimaging studies on patients with NSSI have found that self-processing, a feature of NSSI, is correlated with abnormal brain regions in the DMN, including the PCC, precuneus, and MPFC (35, 36). Although imaging studies on NSSI are limited, existing studies have shown that adults with deliberate self-injury have stronger activity in the PCC than depressed patients without a history of self-injury when performing emotional processing tasks (37). In addition, patients with NSSI have also presented higher neural activity in the MPFC than healthy controls during emotional picture processing (19). Hence, illustrating the underlying relationship between neural function in certain areas of the DMN and NSSI in patients with MDD is important for understanding the neurobiological mechanisms of NSSI behavior.

Guo et al. reported decreased voxel-mirrored homotopic connectivity in certain areas of the visual regions and DMN in patients with first-episode, drug-naive MDD relative to healthy controls (38), which demonstrated that neural alterations in these regions may be connected to the neurobiological mechanisms of MDD. Based on these studies, it is worth further exploring whether there are differences in ALFF and rs-FC in these regions between adolescents with MDD who engage in NSSI and adolescents with MDD only. The ALFF and rs-FC analyses are extensively used and effective rs-fMRI analysis methods, which are generally accepted by the academic field. The ALFF (the specific frequency range is 0.01–0.08 Hz) analysis is used to investigate neural activity by detecting the regional spontaneous fluctuations in brain blood oxygenation level dependent (BOLD) signals (39). The rs-FC analysis was proposed to discover the relevant fluctuations in BOLD signals among distant regions (40). Based on previous studies, we performed ALFF analysis and ALFF-based whole-brain rs-FC analysis to investigate the following hypotheses: (1) the differences in neural activity between adolescents with MDD who engage in NSSI and adolescents with MDD only may be located in certain areas of the visual regions and DMN and (2) the remote rs-FC in the changed regions may be obviously different in adolescents with MDD with and without a history of NSSI.

## Methods

### Subjects

A total of 76 right-handed Han Chinese adolescents (age range: 13–19 years) with a first episode of MDD were recruited from the Department of Psychiatry at the First Affiliated Hospital of Chongqing Medical University. None of the patients took any antidepressants for at least 4 weeks before participating in the study. Subjects with a neurological disorder, history of head trauma with loss of consciousness, history of electroconvulsive therapy, any history of Diagnostic and Statistical Manual IV (DSM-IV) Axis I diagnosis, history of substance or drug addiction/abuse, or contraindications for MRI were excluded. Nine patients who did not meet the criteria for NSSI behavior in the present study were excluded based on clinical interviews. The last NSSI behavior of two of the excluded patients had occurred 6 months previously and seven patients had attempted suicide.

This study was approved by the Research Ethics Committee of the First Affiliated Hospital of Chongqing Medical University. All subjects, accompanied by their parents or legal guardians, were informed about the study in detail, and written informed consent was obtained (signed by a parent or legal guardian for subjects younger than 18 years of age).

### Clinical Assessment

MDD was diagnosed by two senior psychiatrists using the DSM-IV Structured Clinical Interview (SCID) (41) for subjects aged 18 years or older and the Kiddie Schedule for Affective Disorder and Schizophrenia for School-Age Children-Present and Lifetime Version (K-SADS-PL) (42) for subjects younger than 18 years. The 17-item Hamilton Depression Rating Scale (HAMD) was used to assess the severity of MDD owing to its relatively high reliability and validity. In addition, clinical interviews related to NSSI behavior were conducted with all patients with MDD, regardless of whether they had a parent- or self-reported NSSI. Adolescents with MDD who were engaged in NSSI were screened out. The frequency and methods of NSSI in the past 6 months, including skin cutting, scratching, and hair pulling were also obtained. Patients with MDD were divided into the NSSI group (patients with MDD who engaged in NSSI) and MDD group (patients with MDD only) based on their NSSI behavior.

### Acquisition of rs-fMRI Data

MRI images were obtained using a 3T GE Signa HDxt MRI scanner (General Electric Healthcare, Chicago, IL, USA) with an 8-channel head coil. The subjects were instructed to relax with their eyes closed, stay awake, and avoid thinking as much as possible. None of the patients reported falling asleep during scanning. Suitable and comfortable foam pads and earplugs were used to fix their heads to minimize head motion and reduce machine noise, respectively. The EPI pulse sequence parameters were as follows: repetition time (TR), 2,000 ms; echo time (TE), 40 ms; field of view (FOV), 240 × 240 mm^2^; matrix, 64 × 64; flip angle, 90°; slice number, 33; slice thickness/gap, 4.0/0 mm; scanner time, 8 min; and 240 volumes. Three-dimensional T1-weighted MRI images were used for rs-fMRI co-registration. The parameters were also recorded: TR, 24 ms; TE, 9 ms; FOV, 240 × 240 mm^2^; matrix, 256 × 256; flip angle, 90°; and slice thickness/gap, 1.0/0 mm.

### Preprocessing of rs-fMRI Data

The rs-fMRI images were preprocessed using the SPM12-based DPABI software (43) (http://www.restfmri.net) running in MATLAB (MathWorks, Natick, MA, USA). To allow the subjects to adapt to the scanning environment and the magnetization signal to become relatively stable, the first 10 volumes were discarded. The images underwent the following preprocessing steps: (1) slice timing correction, (2) realignment to correct head motion, (3) normalization to the standard Montreal Neurological Institute (MNI) space using the Diffeomorphic Anatomical Registration Through Exponentiated Lie Algebra (DARTEL) tool (44) and resampling to a 3 × 3 × 3 mm resolution. To reduce the effect of physiological noise, a series of nuisance covariates were regressed using the Friston-24 model (45), including the white matter signal, cerebrospinal fluid signal, and head motion parameters. A linear detrend was conducted to reduce the effect of drift in the BOLD signal. Subjects with head translation >3 mm and rotation >3° were removed.

### ALFF Analysis

After preprocessing the data, DPABI software was used to conduct the ALFF analysis. A band-pass filter (0.01–0.08 Hz) was then applied to reduce the influence of lower-frequency drift and high-frequency noise. Each voxel's time series was transformed into the frequency domain using the fast Fourier transform algorithm. After obtaining the power spectrum, the average square root of the power spectrum, namely, the ALFF value, was calculated. The ALFF values were then converted to z-ALFF values using the Fisher's z transformation. The z-ALFF values were spatially smoothed with an isotropic Gaussian kernel with a 4-mm full-width at half-maximum (FWHM). These data were used as the final ALFF values for the statistical analysis.

### Seed Region selection

After the ALFF analysis, the regions with statistical differences in brain activity were regarded as regions of interest (ROI). The seed regions were set in the ROI to further study the integration of the brain function network. The sphere was plotted with the peak voxel of each positive cluster as the center, with a radius of 6 mm, which was considered as the seed region of the rs-FC analysis.

### ALFF-Based Whole-Brain rs-FC Analysis

The DPABI software was used again to study the integration of the brain function network by ALFF-based whole-brain rs-FC analysis. First, after preprocessing, the data were processed using a band-pass filter (0.01–0.08 Hz) and spatially smoothed with a 4-mm FWHM Gaussian kernel. Second, an FC map was generated by calculating the linear correlations between the average time series within each ROI and the rest of the brain voxels. Third, Fisher's r-to-z transformation was conducted to create subject-specific maps, and further statistical analysis was performed.

### Statistical Analysis

To investigate the differences in the demographics and clinical characteristics of the NSSI and MDD groups, the two-sample *t*-test and chi-square test were used to analyze the continuous variables and categorical variables using IBM-SPSS for Windows version 21.0 (IBM Corp., Armonk, NY, USA), respectively. The statistical analysis of ALFF and FC was completed using DPABI software. We used a two-sample *t*-test with age, gender, educational level, and whole-brain gray matter volume as covariates to identify the difference in ALFF between the two groups. The significance level was set at *p* < 0.05. The threshold-free cluster enhancement (TFCE) (46) correction with 1,000 permutations (47) was used to correct for multiple comparisons. For the FC analysis, a one-sample *t*-test was conducted to identify FC abnormalities in each group. The difference in FC between the NSSI and MDD groups was evaluated using a mask created by a one-sample *t*-test statistical map of the two groups. A two-sample *t*-test with age, gender, educational level, and whole-brain gray matter volume as covariates was used to evaluate the significantly distinct FC values in the two groups. During the two-sample *t*-test of FC, the significance level was set at *p* < 0.05. The TFCE (46) correction with 1,000 permutations (47) was used to correct for multiple comparisons. In addition, after extracting the averaged eigenvalues of ALFF and FC with significant inter-group differences, a two-tailed Pearson's correlation analysis with a statistical significance threshold set to *p* < 0.05, was used to investigate the relationship between the abnormal regions and HAMD scores.

## Results

### Demographic Data and Clinical Characteristics

Nine subjects who did not meet the criteria for NSSI behavior in the present study were excluded. The demographics and clinical characteristics of the remaining 67 patients with MDD are shown in Table 1. The age range was 13–19 years. A total of 31 adolescents (8 male and 23 female subjects) with MDD who engage in NSSI with a mean age of 16.13 ± 1.69 years were assessed as the NSSI group. A total of 36 adolescents (9 male and 27 female subjects) with MDD only with a mean age of 16.78 ± 1.48 years were assessed as the MDD group. There were no significant differences in age, gender, and education level between the NSSI and MDD groups (all *p* > 0.05). No obvious differences in HAMD scores were observed between the NSSI and MDD groups (*t* = 0.697, *p* > 0.05). Cutting was the main method of NSSI, and a few individuals used multiple NSSI methods.

**Table 1:** Demographics and clinical characteristics of adolescents with MDD who engage in NSSI and adolescents with MDD only.

**Characteristics**		**NSSI**		**MDD**		**Statistical test**	***P-*value**
		**(*****n*** **=** **31)**		**(*****n*** **=** **36)**			
		**Mean**	**SD**	**Mean**	**SD**		
Age (years)		16.13	±1.69	16.78	± 1.48	*t* = −1.679	0.098
Gender (Male: Female)		8:23		9:27		χ^2^ = 0.006	0.940
Education level (years)		9.98	± 1.91	10.47	± 1.59	*t* = −1.180	0.242
HAMD score		21.19	± 3.27	20.61	± 3.52	*t* = 0.697	0.488
**NSSI**							
Frequency							
	1–5 acts	13 (41.94%)					
	6–10 acts	11 (35.48%)					
	11–20 acts	5 (16.13%)					
	21^+^	2 (6.45%)					
Ways	Cutting	25					
	Interfere with wound	3					
	Sever scratching	3					
	Hair pulling	1					
	Banging/hitting self	5					

*MDD, major depressive disorder; NSSI, non-suicidal self-injury; HAMD, Hamilton Depression Rating Scale. The chi-square test was used for gender comparisons. The two-sample t-test was used to compare age, education level, and HAMD score*.

### ALFF Results

**Figure 1:**
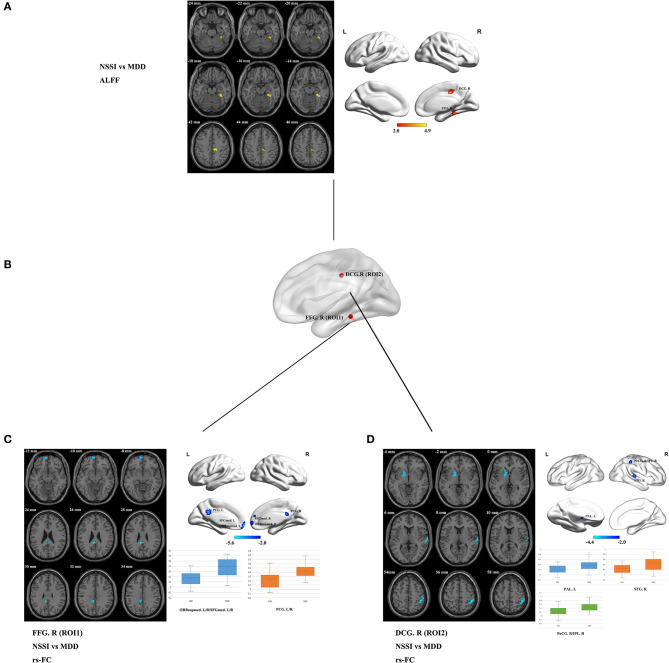
**(A)** Brain regions showing significant differences in ALFF values in the NSSI group compared with the MDD group (*p* < 0.05, 1,000 permutations, TFCE corrected). Regions where the ALFF values have increased are shown in red. The color bar indicates the *T-*value. **(B)** The locations of the seed regions are based on the ROI (ROI1 and ROI2). **(C)** Brain regions showing significant differences in ROI1 (FFG. R)-based rs-FC in the NSSI group compared with the MDD group. Regions where the rs-FC has decreased are shown in blue (*p* < 0.05, 1,000 permutations, TFCE corrected). Box plot showing the comparisons of ROI1 (FFG. R)-based rs-FC values between the NSSI and MDD groups. The center line, the lower bound of the box, and the upper bound of the box represent the median value, the 25th percentile, and the 75th percentile, respectively, and the whiskers represent the maximum and minimum values. **(D)** Brain regions showing significant differences in ROI2 (DCG. R)-based rs-FC in the NSSI group compared with the MDD group. Regions where the rs-FC has decreased are shown in blue (*p* < 0.05, 1,000 permutations, TFCE corrected). Box plot showing the comparisons of ROI2 (DCG. R)-based rs-FC values between the NSSI and MDD groups. The center line, the lower bound of the box, and the upper bound of the box represent the median value, the 25th percentile, and the 75th percentile, respectively, and the whiskers represent the maximum and minimum values. ALFF, amplitude of low-frequency fluctuation; MDD, major depressive disorder; NSSI, non-suicidal self-injury; rs-FC, resting-state functional connectivity; ROI, region of interest; TFCE, threshold-free cluster enhancement. FFG. R, right fusiform gyrus; DCG. R, right median cingulate and paracingulate gyri; ORBsupmed. L/R, bilateral medial orbital of the superior frontal gyrus; SFGmed. L/R, bilateral medial superior frontal gyrus; PCG. L/R, bilateral posterior cingulate gyrus; PAL. L, left pallidum; STG. R, right superior temporal gyrus; PoCG. R, right postcentral gyrus; IPL. R, right inferior parietal lobule.

The brain regions with statistical differences in ALFF values between the NSSI and MDD groups are displayed in Table 2, Figure 1A. Compared to the MDD group, the NSSI group showed significantly increased ALFF in the right FFG (FFG. R) (MNI peak voxel coordinates: 27, −36, −15), and the right median cingulate and paracingulate gyri (DCG. R) (MNI peak voxel coordinates: 12, −24, 39) (*P* < 0.05). There was no evidence of brain regions with decreased ALFF in the NSSI group. The clusters that presented significantly increased ALFF in the NSSI group relative to the MDD group were used as ROI for further rs-FC analysis. We defined a sphere with a radius of 6 mm centered on the peak voxel coordinates of the ROI as the seed region for further analysis, as shown in Figure 1B.

**Table 2:** Brain regions showing significantly decreased ALFF values in the adolescents with MDD who engage in NSSI compared with the adolescents with MDD only.

**Cluster**	**Region**	**Side**	**Cluster size (voxels)**	**MNI coordinate**			**Peak intensity**
				**x**	**y**	**z**	
NSSI > MDD							
Cluster 1 (ROI1)	FFG	R	52	27	−36	−15	4.9059
Cluster 2 (ROI2)	DCG	R	29	12	−24	39	3.9877

*ALFF, amplitude of low-frequency fluctuation; MDD, major depressive disorder; NSSI, non-suicidal self-injury; MNI, Montreal Neurological Institute; R, right; FFG, fusiform gyrus; DCG, median cingulate and paracingulate gyri*.

### ALFF-Based rs-FC Analysis

Based on the ALFF analysis, spherical ROI with a radius of 6 mm centered on the peak voxel coordinates in the FFG. R and DCG. R as seed regions (ROI1 and ROI2, respectively) were obtained to conduct the subsequent ALFF-based rs-FC analysis (see Table 2, Figure 1B). Compared with the MDD group, the rs-FC of the FFG. R-bilateral medial orbital of the superior frontal gyrus (ORBsupmed. L/R)/bilateral medial superior frontal gyrus (SFGmed. L/R) and FFG. R-bilateral posterior cingulate gyrus (PCG. L/R) was decreased in the NSSI group (Table 3, Figure 1C). In addition, the rs-FC of the DCG. R-left pallidum (PAL. L), DCG. R-right superior temporal gyrus (STG. R), and DCG. R-right postcentral gyrus (PoCG. R)/right inferior parietal lobule (IPL. R) was significantly reduced in the NSSI group compared to the MDD group (Table 3, Figure 1D). There was no evidence of brain regions with increased rs-FC in the NSSI group.

**Table 3:** Brain regions showing significantly decreased ROI1-based rs-FC and ROI2-based rs-FC in adolescents with MDD who engage in NSSI compared with the adolescents with MDD only.

**Cluster**	**Region**	**Side**	**Cluster size (voxels)**	**MNI coordinate**			**Peak intensity**
				**x**	**y**	**z**	
**ROI1**							
NSSI < MDD							
Cluster 1	ORBsupmed/SFGmed	L/R	130	0	63	18	−5.5762
Cluster 2	PCG	L/R	40	−9	−39	27	−5.4528
**ROI2**							
NSSI < MDD							
Cluster 1	PAL	L	61	−12	3	−3	−3.8052
Cluster 2	STG	R	51	63	−33	3	−4.0349
Cluster 3	PoCG/IPL	R	85	45	−39	57	−4.4425

*MDD, major depressive disorder; NSSI, non-suicidal self-injury; rs-FC, resting-state functional connectivity; MNI, Montreal Neurological Institute; ROI, region of interest; L, left; R, right; ORBsupmed, medial orbital of the superior frontal gyrus; SFGmed, medial superior frontal gyrus; PCG, posterior cingulate gyrus; PAL, pallidum; STG, superior temporal gyrus; PoCG, postcentral gyrus; IPL, inferior parietal lobule*.

### Correlation of ALFF/FC With HAMD Scores

Average eigenvalues of ALFF in the FFG. R and DCG. R and FC from these two ROI to the other regions (see Table 3) were extracted to further evaluate their association with HAMD scores. There was no significant association between HAMD scores and ALFF or FC values in the NSSI and MDD groups (*p* > 0.05).

## Discussion

Rs-fMRI, a non-invasive imaging method, is widely used to study anomalous neural function in individuals with psychiatric disorders. In the current study, 67 adolescents with MDD were registered, and 31 patients had NSSI behavior according to clinical interviews. Abnormal neural function in certain areas of the visual regions and DMN in the NSSI group relative to the MDD group was detected using ALFF analysis. Our results showed that, compared to the MDD group, the brain regions with increased ALFF in the NSSI group were located in the FFG. R and DCG. R, but there was no evidence of brain regions with decreased ALFF in the NSSI group. After ALFF analysis, significantly decreased connectivity of the FFG. R-ORBsupmed. L/R/SFGmed. L/R, FFG. R-PCG. L/R, DCG. R-PAL. L, DCG. R-STG. R, and DCG. R-PoCG. R/IPL. R in the NSSI group relative to the MDD group was also detected using whole-brain ALFF-based rs-FC analysis. These main findings are consistent with our hypotheses, suggesting that the abnormal patterns within certain areas of the visual regions and DMN in adolescents with MDD who engage in NSSI and adolescents with MDD only may be correlated with NSSI behavior.

In the present study, the brain areas with increased ALFF in adolescents with MDD who engaged in NSSI were located in the FFG. R and DCG. R. The FFG. R is involved in the composition of visual regions that play a vital role in the perception of emotion during facial stimuli tasks (48). Enhanced neural activity in response to sad facial expression in the FFG. R was observed in patients with MDD compared to healthy controls, while a decreased response was also exhibited in the bilateral FFG in response to happy facial stimuli, which was inversely correlated with depression severity (49). In addition, Chan et al. demonstrated that increased activity in the FFG. R is associated with a high risk of depression (50). These studies suggest abnormal neural activity in the FFG. R may be associated with the severity of depression or susceptibility to depression. The alteration of the FFG. R between the NSSI and MDD groups suggested that NSSI behavior may be a potential measure of the severity of the MDD. However, there was no significant correlation between the HAMD scores and ALFF alterations in the FFG. R in our study. Therefore, it is necessary to expand the sample size for further studies. Contrary to the above results, another rs-fMRI study found significantly decreased ALFF in the FFG. R among patients with MDD compared to healthy controls (51). Skokauskas et al. reported similar results after analyzing abnormal neural activity in patients with MDD only, patients with MDD and sexual abuse, and healthy controls using task-based fMRI (52). The inconsistent results of the aforementioned studies may have been due to differences in sample size, imaging parameters, task modes, and effect of MDD symptoms. Our work focused on rs-fMRI in adolescents with MDD who engage in NSSI, especially in certain areas of the visual regions and DMN. Individuals with NSSI behavior usually have an aberrant ability to recognize, understand, process, and express emotion (53). NSSI is a common strategy among adolescents to regulate negative emotions (54). Emerging studies have demonstrated that greater difficulty with emotional awareness (55) and regulation (56) in individuals who engage in NSSI may be connected to NSSI behavior. Adolescent inpatients who engage in NSSI showed greater deficits in emotional face recognition than healthy controls (57), which suggests that deficits in emotional face recognition may be correlated with dysfunctional visual regions, including the FFG. R. We found increased ALFF values in the FFG. R in adolescents with MDD who engaged in NSSI relative to adolescents with MDD only. This result is in line with a report that, compared to healthy controls and subjects with depression only, NSSI youth exhibited enhanced neural activity in the FFG (23). The differences in neural activity of the visual areas such as the FFG between the two groups imply that it may potentially be involved in the neurobiological process of NSSI in adolescents with MDD. However, further research is required in the future. The NSSI group also showed an increased ALFF in the DCG. R. The DCG is involved in the composition of the cingulate gyrus, which is the key node in the DMN and plays an important role in information transmission and cognitive processing. The DMN is significantly associated with episodic memory and rumination processing of depressive symptoms (27, 58). However, there was no prior rs-fMRI study on DCG and MDD or NSSI. Previous studies have focused more on PCC, one of the major subdivisions of the DMN. Aberrant neural activity in the PCC and PCC-based FC in patients with MDD has been widely reported (59, 60). FC in the PCC has been shown to be correlated with rumination in MDD (30). In the present study, the rs-FC between the FFG. R and PCG. L/R in the NSSI group compared to the MDD group was also decreased, which is similar to previous evidence showing that, compared to healthy controls, patients with MDD had significantly reduced rs-FC between the PCC and FFG (61). Taken together, these results indicate that differences in brain activity between the NSSI and MDD groups might exist at multiple levels including visual information processing, the perception of emotions, and the integration of affective and cognitive information, and to some extent, it could provide an explanation for the occurrence of NSSI behavior in adolescents with MDD.

Based on the changes in the ALFF in the FFG. R, rs-FC from the FFG. R to the whole brain was analyzed in the present study. To the best of our knowledge, the connectivity patterns of the FFG. R with other brain areas in adolescents with MDD who engage in NSSI has not been previously reported. Our findings showed that, compared to patients with MDD only, significantly decreased connectivity of the FFG. R-ORBsupmed. L/R/SFGmed. L/R and FFG. R-PCG. L/R was detected in adolescents with MDD who were engaged in NSSI. There was no evidence of brain regions with increased rs-FC in the NSSI group. These findings support our hypotheses that the differences in neural activity between adolescents with MDD who engage in NSSI and adolescents with MDD only might be located in certain areas of the visual regions and DMN, and the remote rs-FC in the changed brain regions might be significantly different. The frontal cortex is a pivotal part of the fronto-striatal circuit, which involves the superior frontal gyrus (ORBsupmed and SFGmed) and PAL. Previous studies have demonstrated that the superior frontal gyrus is associated with cognitive processing involved in executive function and memory retrieval in patients with MDD (62). Moreover, a limited number of studies have shown that individuals with NSSI behavior show hyperactivation in frontal areas, including the ORBsupmed and SFGmed, striatum, and STG (63, 64); however, no research on FC in these regions in patients with NSSI has been reported. An fMRI study suggested that, compared to healthy controls, patients with MDD showed decreased FC in the superior frontal gyrus, which is similar to our results (65). Given the association between the frontal cortex and emotional regulation and impulsive control (66), the decreased FC in the superior frontal gyrus in adolescents with MDD who engage in NSSI prompted us to carefully consider the effect of the superior frontal gyrus on NSSI behavior.

Decreased FC from the DCG. R to the PAL. L, STG. R, and PoCG. R/IPL. R was also examined in adolescents with MDD who were engaged in NSSI. The PAL, which is not only a component of the striatum but also a transmission node connecting the PFC and amygdala, participates in the mediation of reward processing and anhedonia in MDD (67). Reward processing is related to the feeling of “relief” in patients with NSSI. The association between striatum activity and relief was greater in NSSI youth than that in non-NSSI youth, indicating that the striatum might be involved in the neurobiological process of NSSI behavior (63). Interestingly, a previous study suggested that adolescents with NSSI thoughts also showed enhanced striatal activity during the monetary reward task relative to those without any NSSI thoughts (68), which could be another evidence of a link between the striatum and NSSI. However, there has been no research on FC in the striatum of individuals with NSSI. In accordance with prior research, the present study further confirmed that the abnormal connection in the PAL may participate in the NSSI behavior of adolescents with MDD. Furthermore, accumulating evidence has demonstrated that the temporal lobe is linked to human emotional and mental activities. In addition to auditory processing, the STG is also implicated in emotional processing and social cognition in patients with MDD (69, 70). Guo et al. found that, compared to healthy controls, drug-naive patients with MDD had significantly decreased FC in the STG (71). In addition, an enhanced BOLD response in the STG was also demonstrated in individuals with NSSI behavior (63). The rs-FC was also reduced in the PoCG, which participates in somatosensory processing, in youth with MDD relative to healthy controls (72). However, to our knowledge, there are no reports on the changes in FC in the STG and PoCG in NSSI individuals. In the present study, compared to the adolescents with MDD only, the adolescents with MDD who engaged in NSSI demonstrated significantly reduced connectivity of the DCG. R-STG. R and DCG. R-PoCG. R. Taken together, these differences have the potential to be used as specific brain regions to help differentiate patients with MDD who engage in NSSI from all patients with MDD and contribute to further studies on the neurobiological mechanism of NSSI in the future. In addition, these results also imply that NSSI behavior may be a potential measure of the severity of MDD. However, further studies are required.

### Limitations

This study has limitations that merit mention. First, the history of NSSI behavior in adolescents with MDD came from a parent or self-report and further clinical interviews, so it is probable that subjects may engage in self-injury behavior with the intention of ending their life but deny it owing to a sense of shame. However, to date, we have not been able to determine their purpose. Second, owing to the cross-sectional design, the changes in ALFF in the FFG. R and DCG. R or in rs-FC from the FFG. R and DCG. R to other related regions did not demonstrate that it was a state or trait effect in adolescents with MDD who engage in NSSI; therefore, it is necessary to conduct a longitudinal study in future research. Third, to explore the neurobiological changes in adolescents with MDD who engage in NSSI, we used adolescents with MDD only as a control, but, healthy controls may be needed to determine whether there are differences between patients with MDD and healthy people in the same brain region. Fourth, the present study lacks an objective assessment of the IQ of all subjects, although IQ scores are more likely to be correlated with the frontoparietal network (73). Finally, the sample size was relatively small. It is necessary to increase the sample size for further rs-fMRI studies on adolescents with MDD who engage in NSSI.

## Conclusion

In conclusion, the current study has demonstrated that, in comparison with adolescents with MDD only, significantly enhanced local brain activity in the FFG. R and DCG. R and the significantly reduced rs-FC of the FFG. R-ORBsupmed. L/R/SFGmed. L/R, FFG. R-PCG. L/R, DCG. R-PAL. L, DCG. R-STG. R, and DCG. R-PoCG. R/IPL. R was detected in adolescents with MDD who engage in NSSI. These results are in line with our hypotheses that the differences in local neural activity between adolescents with MDD who engage in NSSI and adolescents with MDD only may be located in certain areas of the visual regions and DMN, and the remote rs-FC based in the changed regions in adolescents with MDD with and without a history of NSSI may be obviously different. These differences may be helpful in understanding the neural alterations of adolescents with MDD who engage in NSSI and could provide a new perspective for further study on the neurobiological mechanism of NSSI behavior in adolescents with MDD.

## Data Availability Statement

The original contributions presented in the study are included in the article/supplementary material, further inquiries can be directed to the corresponding author/s.

## Ethics Statement

The studies involving human participants were reviewed and approved by Ethics Committee of the First Affiliated Hospital of Chongqing Medical University. Written informed consent to participate in this study was provided by the participants' legal guardian/next of kin.

## Author Contributions

QH designed the research, collected samples, analyzed data, and wrote the original of manuscript. MX collected samples, supervised, and analyzed data. MA, WW, and JCh supervised data and conducted quality control. LH collected samples and gave some advice. JCa supervised the research, gave some advice, and revised the manuscript. MW collected samples. LK supervised the research, provided funding, and gave some advice. All authors contributed to the article and approved the submitted version.

## Conflict of Interest

The authors declare that the research was conducted in the absence of any commercial or financial relationships that could be construed as a potential conflict of interest.
